# Moderating Effects of Racial Segregation on the Associations of Cardiovascular Outcomes with Walkability in Chicago Metropolitan Area

**DOI:** 10.3390/ijerph192114252

**Published:** 2022-10-31

**Authors:** Hao Huang

**Affiliations:** Department of Social Sciences, Illinois Institute of Technology, Chicago, IL 60616, USA; hhuang48@iit.edu

**Keywords:** social segregation, cardiovascular health outcomes, walkability, diabetes, minority population, Chicago

## Abstract

Cardiovascular diseases (CVDs), as the leading cause of death in the U.S., pose a disproportionate burden to racial/ethnic minorities. Walkability, as a key concept of the built environment, reflecting walking and physical activity, is associated with health behaviors that help to reduce CVDs risk. While the unequal social variation and spatial distribution inequality of the CVDs and the role of walkability in preventing CVDs have been explored, the moderating factors through which walkability affects CVDs have not been quantitatively analyzed. In this paper, the spatial statistical techniques combined with the regression model are conducted to study the distribution of the CVDs’ health outcomes and factors influencing their variation in the Chicago metropolitan area. The spatial statistical results for the CVDs’ health outcomes reveal that clusters of low-value incidence are concentrated in the suburban rural areas and areas on the north side of the city, while the high-value clusters are concentrated in the west and south sides of the city and areas extending beyond the western and southern city boundaries. The regression results indicate that racial segregation reduced the positive association between health outcomes and walkability, although both racial segregation and walkability factors were positively associated with CVDs’ health outcomes.

## 1. Introduction

Cardiovascular diseases (CVDs), the leading cause of death in the U.S., poses a disproportionate burden to racial/ethnic minorities [1]. Walkability as a key concept of the built environment indicates a neighborhood’s capacity to support individuals’ lifestyle behaviors such as walking and physical activity [2], which is associated with health behaviors helping reduce CVDs risk [3]. Increasing opportunities for physical activity in neighborhoods are a part of public health efforts to reduce CVDs incidences since favorable built environmental conditions in the neighborhood are associated with exercise behaviors and reduced risk for CVDs [4,5,6,7]. Walking environments can increase walking, biking, and physical activity [8,9,10] to reduce disease and improve overall health [5,11,12,13]. Thus, walkability and safety are essential features of the built environment for people to exercise or walk to engage in physical activity for health, which justifies the importance of studying the built environmental correlations of pedestrian walkability in preventing CVDs [14,15].

Racial/ethnic disparities in walkability exist within the city [16], so improved walkability may have differential effects on CVDs for racial/ethnic subpopulations. Previous studies have assessed neighborhood walkability using various measurements such as street connectivity, residential density, and land-use mix [7,15,17,18]. Research has associated increased neighborhood walkability with decreased risk factors of CVDs [3,19] such as hypertension [14]. A deeper understanding of the interaction of racial segregation with the relationship between neighborhood walkability and CVDs is needed to develop initiatives and inform policymakers and urban designers on redesigning cities and suburbs to improve public health.

There has been an increase in the literature on the role of walkability in preventing CVDs since increased poor connectivity during the past decades has resulted in creating obesogenic environments in developed economies [14]. However, very few studies have quantitatively analyzed specific moderating factors through which walkability affects CVDs. Previous studies showed that neighborhood walkability is negatively related to CVDs risk factors, such as diabetes [5] and hypertension, especially for low-income individuals [14]. In addition, studies suggest that the magnitude of an association between walkability and CVDs may be quite small, depending on whether walkability measures responsive to the local environment can powerfully predict CVDs [19]. Moreover, census tracts with significant minority concentrations had high levels of poor walkability and premature CVDs mortality [3].

The residential segregation by social and physical environments contributes to CVD risk [20,21,22]. On one hand, racial/ethnic residential segregation may represent an important determinant influencing CVD risk, particularly in minorities [23,24,25,26]. During the 20th century, residential segregation increased, with a long period of institutional discrimination leading to constrained opportunities for African American and Hispanic residential mobility [27,28]. The increased residential segregation results in limited access to health resources, so segregation has been considered a fundamental cause of racial health disparities [29,30,31]. Thus, higher segregation was related to worse CVD risk [32,33,34]. On the other hand, walkability as a neighborhood feature related to physical activity contributes to the disparity in CVD risk [21]; mixed uses, more destinations, and greater connectivity are related to higher walkability [35,36]. Residential segregation could influence health by limiting the opportunities to access health-promoting resources and amenities [37]. Thus, there may be an association between residential segregation and neighborhood walkability. However, the moderating effects of racial segregation on the relationship between neighborhood walkability and CVDs remain unclear in the context of racially segregated cities and regions.

Thus, to the author’s knowledge, only one study has investigated the interaction effect of walkability and race on CVDs: it is found that Black premature CVD deaths accounted for a disproportionate number of the premature CVD deaths in all census tracts in Atlanta [3], but no significant effect of interaction between walkability and Black population percentage on CVDs was found. However, Chicago is much more racially segregated than Atlanta. According to the Black-White dissimilarity index, Chicago is ranked fifth in the Diversity and Disparities project by Brown University [38]. Cities and regions have their own sociodemographic, historical, and environmental contexts, so racial segregation may have different effects on the interaction between the built environment and CVDs, in terms of varied contexts.

To fill this research gap, the present study employs spatial statistics to understand the spatial patterns of CVDs, walkability, and racial segregation in the Chicago metropolitan area. Specifically, local Moran’s *I* statistics are conducted to examine clusters and outliers of CVDs, walkability, and racial segregation, while Getis-Ords *G* statistics are conducted to examine their hotspots and coldspots. This study uses Ordinary Least Squares (OLS) regression combined with spatial regression models to explore the moderating effects of racial segregation on the relationship between walkability and CVDs.

## 2. Materials and Methods

### 2.1. Data Sources

The health outcomes data are from the 500 Cities Project, a collaboration between the Centers for Disease Control and Prevention, the Robert Wood Johnson Foundation, and the CDC Foundation [39]. This project reports census-tract-level data and uses small-area-estimation methods to obtain 29 chronic disease measures for 500 cities in the United States between 2016 and 2019. Theil’s H index of racial segregation is from CDC and PolicyMap [40]. The White percentage, Black percentage, and Asian percentage data are from the American Community Survey 2019 [41]. The minority population percentage data are from the Environmental Justice Mapping and Screening Tool provided by the U.S. Environmental Protection Agency (EPA) [42]. The walkability variable data are from the U.S. EPA Smart Growth Program [43]. The data from these different sources are merged based on the census-tract identification number. The urban-area shapefile data are from the United States Census Bureau [44]. The Census Bureau delineates urban areas that represent densely developed territories, encompassing residential, commercial, and other nonresidential urban land uses. In general, urban areas consist of areas of high population density and urban land use, resulting in a representation of the urban footprint. There are two types of urban areas: urbanized areas that contain 50,000 or more people and urban clusters that contain at least 2500 people but fewer than 50,000 people [45].

### 2.2. Health Outcomes

Five health outcomes among adults aged equal to or older than 18 years old in this study are hypertension, high cholesterol, diabetes, coronary heart disease, and stroke. Hypertension, high cholesterol, diabetes, coronary heart disease, and stroke are measured as the respective prevalence of high blood pressure, high cholesterol, diabetes, coronary heart disease, and stroke among adults aged equal to or older than 18 years. The prevalence is calculated by respondents aged equal to or older than 18 years who report ever having been told by health professionals that they have each health outcome as the numerator and the respondents aged equal to or older than 18 years as the denominator.

### 2.3. Explanatory Variables Measuring Factors That Impact Health Outcomes

Explanatory variables are used to retrieve variables measuring racial segregation and neighborhood walkability. The racial segregation variables are the racial segregation Theil index and different racial/ethnic population percentages including the White percentage, the Black percentage, the Asian percentage, and the Minority percentage.

The Theil index is widely used to measure racial segregation in the literature [46,47,48] since it is a comprehensive measure of segregation related to inequality measures [49]. Theil’s H index of racial segregation is calculated at the census-tract level using the U.S. Census Bureau’s 2010 Decennial Census estimates. This index combines the following eight racial/ethnic categories provided by the U.S. Census Bureau: Hispanic, White, African American, American Indian or Alaska Native, Asian, Native Hawaiian or Pacific Islander, some other race, and two or more races. This index estimates the extent to which racial and ethnic groups are evenly distributed in a census tract area, which ranges from 0 to 1. Values approaching 0 suggest even distribution and less segregation, while values approaching 1 suggest non-uniform distribution and more segregation.

To be consistent with the spatial scale of Theil’s H index analysis, the data of the White percentage, the Black percentage, and the Asian percentage measure the population share for each race at the census-tract level by (1) retrieving the data at the block level from the American Community Survey 2019 [41] and by (2) aggregating them at the census-tract level by calculating the median value in a census tract. The minority percentage data are from the Environmental Justice Mapping and Screening Tool provided by the U.S. EPA [42], which is the percentage of all people other than non-Hispanic white-alone individuals.

The walkability variable is the national walkability index from the Smart Location Database (SLD), a publicly available data product and service provided by the U.S. EPA Smart Growth Program [43]. This index combines the following four SLD measures into a composite index: (1) employment and household entropy; (2) static eight-tier employment entropy; (3) street intersection density; and (4) distance to transit stops. These four measures represent different characteristics of walkability: employment and household entropy and the static eight-tier employment entropy were both used as proxies for land-use mix; street intersection density and distance to transit stops represent the street connectivity and access to public transit. The index value is ranged between 1 (lowest walkability) and 20 (highest walkability).

### 2.4. Spatial Statistics and Regression Modeling with Interaction Term

**Figure 1:**
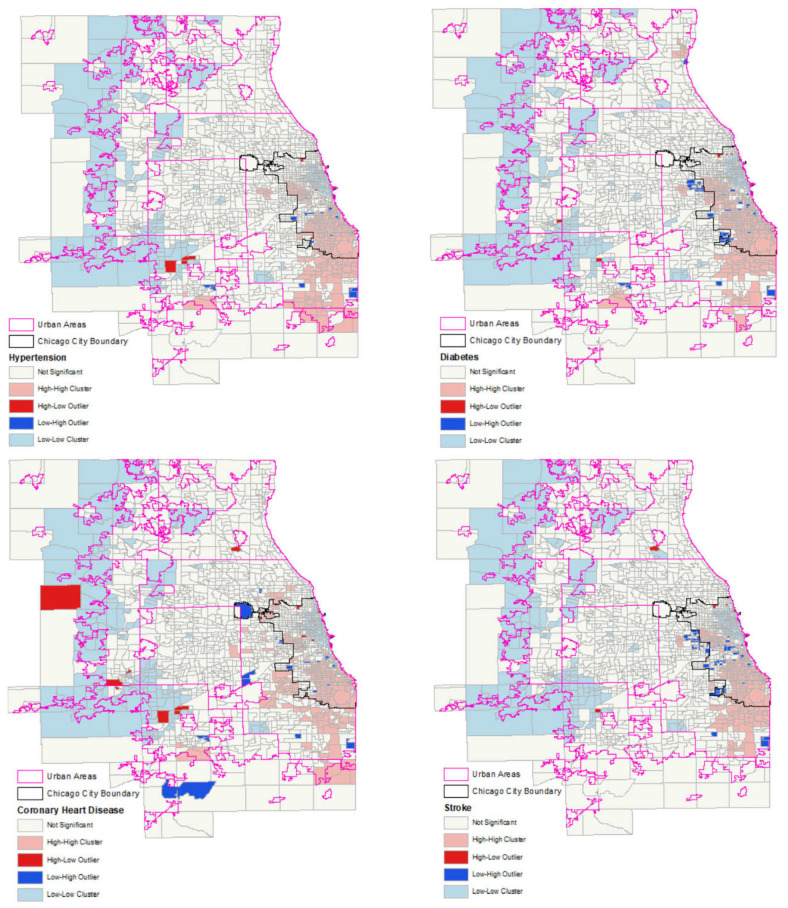
The clusters and outliers of hypertension, diabetes, coronary heart disease, and stroke.

**Figure 2:**
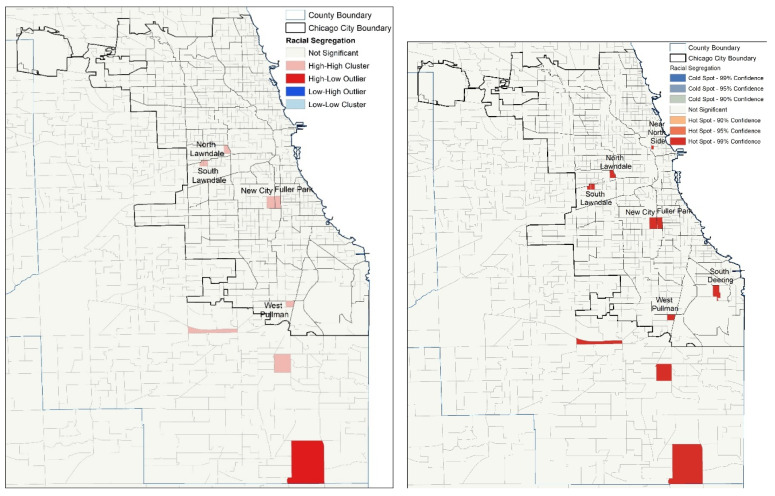
Clusters and outliers of racial segregation index (**left**) and hotspots of racial segregation index (**right**). Since there are no clusters, outliers, hotspots, and coldspots beyond Cook County, the map only shows the spatial extent of Cook County instead of the Chicago metropolitan area.

**Figure 3:**
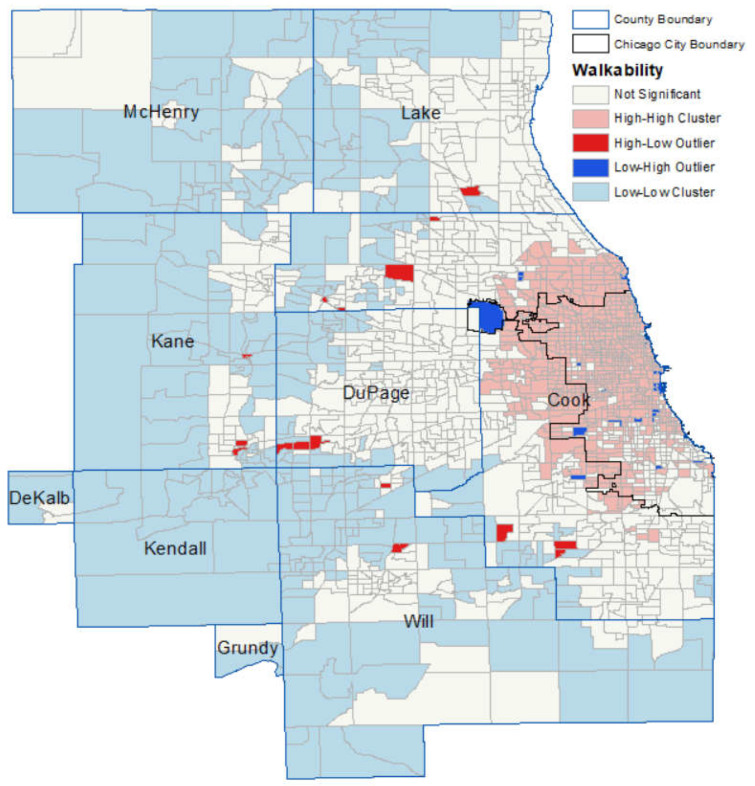
Clusters and outliers of the national walkability index.

Spatial statistics, including local Moran’s *I* and Getis-Ord *G* statistics, were used to detect spatial clusters, outliers, hotspots and coldspots of health outcomes, racial segregation, and walkability index. Spatial clusters of features with high or low values are identified, which are, respectively, labeled as the high-high cluster and low-low cluster in the legend in Figure 1, Figure 2 and Figure 3. Spatial outliers of features with high values surrounded by low values, labeled as the high-low outlier in the legend in Figure 1, Figure 2 and Figure 3, or with low values surrounded by high values, labeled as the low-high outlier in the legend in Figure 1, Figure 2 and Figure 3, are identified. ArcGIS is used to calculate and map Moran’s *I* and Getis-Ord *G* statistics.

The OLS regression models, with interaction terms between racial segregation and walkability variables, were performed to investigate the relationship between CVDs, walkability, and racial segregation. The clusters and outliers of hypertension, diabetes, coronary heart disease, and stroke in Figure 1 show evidence of strong spatial autocorrelations of cardiovascular health outcomes, so an OLS regression model was insufficient to explain the correlates between CVDs, walkability, and racial segregation. Therefore, this research employed spatial regression models in addition to the OLS model to improve prediction by reducing spatial autocorrelation. Two types of spatial regression models conducted in this research are the spatial lag model (SLM) and the spatial error model (SEM). SLM is appropriate for the assessment of spatial interaction, which is interpreted as substantive spatial dependence. Comparatively, SEM is appropriate for the correction of the potentially biasing influence of the spatial autocorrelation, due to the use of spatial data (no matter whether the model is spatial or not), which is interpreted as nuisance dependence [50]. The spatial dependence of the SLM was captured by spatial spillover effects and spatial error correlation effects, while the spatial dependency of the SEM was captured only by spatial error correlation. According to diagnostics for spatial dependence and heteroskedasticity shown in Table 1, both the Lagrange Multiplier Lag statistics and Lagrange Multiplier Error statistics are significant for hypertension, diabetes, and stroke; the Lagrange Multiplier Lag statistic is significant for high cholesterol, and the Lagrange Multiplier Error statistic is significant for coronary heart disease. Thus, SLMs and SEMs are conducted for hypertension, diabetes, and stroke; SLM is conducted for high cholesterol; SEM is conducted for coronary heart disease. Stata and GeoDa are used to perform regression models.

## 3. Results

### 3.1. Descriptive Characteristics of Health Outcomes, Racial Segregation, and Walkability

Table 2 shows the descriptive statistics of independent variables. In terms of the number of cases per 10,000 persons, high cholesterol has been ranked as the highest among the five health outcomes, with 261 cases in each census tract on average. The other four health outcomes, ranked from the highest to the lowest, are sequantially 94 hypertension cases, 35 diabetes cases, 17 coronary heart disease cases, and 11 stroke cases, in each census tract. High cholesterol has the highest average incidence of CVDs, which is almost three times as many as the second-highest average incidence of CVDs, hypertension, and is almost 25 times as many as the lowest average incidence of CVDs, stroke. The health outcomes, ranked from the largest to the smallest variances, are sequentially hypertension, high cholesterol, stroke, diabetes, and coronary heart disease. In addition, high cholesterol and hypertension variances show that they were most affected by geographical factors.

Based on the average racial/ethnic population percentage, the white population accounts for the largest percentage of the total population with 57% in each census tract on average; the black population accounts for 19% of the total population in each census tract; the Asian population accounts for 6% of the total population. In terms of the ratio of the standard deviation to the mean, the black population has the largest variance, showing the largest racial segregation in potential.

### 3.2. Spatial Statistics of Health Outcomes, Racial Segregation, and Walkability

Figure 1 shows that hypertension, diabetes, coronary heart disease, and stroke have similar cluster patterns: the low-value clusters, labeled as low-low clusters in the legend of the map for each health outcome, occupy the nonurban areas and areas on the north side of the city, while the high-value clusters, labeled as high-high clusters in the legend of the map for each health outcome, occupy areas on the west and south sides of the city and areas extending beyond the western and southern city boundaries, although there are slight differences in the high-value clusters among health outcomes. The high-value clusters for hypertension, diabetes, and stroke extend into the areas beyond the southern boundary of the city area, while the high-value clusters for coronary heart disease extend into the areas beyond both the western and southern boundaries of the city area.

Figure 2 shows the clusters, outliers, and hotspots of the racial segregation index. There are eight high-value clusters of the racial segregation index, labeled as high-high clusters in the legend of the map. Six of them are in the communities of North Lawndale, South Lawndale, New City, Fuller Park, and West Pullman on the west and south sides of the city. Another two high-value clusters are in the areas beyond the southern boundary of the city area within Cook County. One high-low outlier is the high racial segregation area surrounded by the low racial segregation areas in the south suburb at the boundary of Cook County, labeled as the high-low outlier in the legend of the map. In addition, there are 11 hotspots of the racial segregation index, labeled as the hot spot with 99% confidence in the legend of the map. Eight of them are in the city area: one of them is in the Near North Side community on the north side, while seven of them are in the communities of North Lawndale, South Lawndale, New City, Fuller Park, West Pullman, and South Deering in the west and south sides. Three of them are in the south suburbs of Cook County.

The west and south sides of the city area have both high-value clusters and hotspots of racial segregation index shown in Figure 2 and are also the high-value clusters of hypertension, diabetes, coronary heart disease, and stroke shown in Figure 1. At the same time, the north side of the city area has the low-value clusters of these health outcomes shown in Figure 1. The similarity of clusters patterns of racial segregation index and health outcomes on the west and south sides shows the potential association between racial segregation and health outcomes, which is examined in the regression models in Section 3.3

Figure 3 shows the spatial statistics results of the national walkability index. In general, the city of Chicago and surrounding northern and western areas have the high-value clusters of the national walkability index, while the suburban (the suburban area is the area between the principal city, Chicago, and the nonurban areas in the Chicago metropolitan area) and nonurban areas have the low-value clusters of the national walkability index. Low-value clusters of walkability index are in the suburbs with sporadic high-low outliers, which are the high walkability areas surrounded by the low walkability areas located on the side close to the city area. In addition, high-value clusters of walkability index are in the city area and areas adjacent to the northern and western boundaries of the city area. So, low-value clusters are mainly located in the majority or entire areas of McHenry County, Kane County, DeKalb County, Kendall County, Grundy County, and Will County. Moreover, high-value clusters are exclusively in Cook County: almost the entire areas on the north and west sides of the city area and areas extending beyond the north and west sides of the city boundary are high-value clusters, while there are only a few high-value clusters on the south side. Besides, a few outlier areas with a high-value index surrounded by census tracts with a low-value index are sporadically located adjacent to the low-value clusters on the side close to the city area. Similarly, a few opposite outlier areas with a low-value index surrounded by census tracts with a high-value index are sporadically located adjacent to the boundary of the high-value clusters. In addition, there are no hotspots and coldspots of walkability. The concentration of high-value clusters of walkability index on the north and west sides, combined with the similar patterns of racial segregation index and health outcomes, shows the potential effects of the association of walkability with racial segregation and health outcomes, which is examined in the regression models in Section 3.3

### 3.3. Regression Models

Table 3 shows the regression results describing the relationships between five health outcomes, racial segregation, and walkability. In the model, there are no associations between the racial segregation index and health outcomes. For different racial/ethnic groups, both the White population percentages and the Black population percentages have positive associations with five health outcomes (i.e., hypertension, high cholesterol, diabetes, coronary heart disease, and stroke); the Asian population percentages have positive associations with the high cholesterol; and the Minority population percentages have positive associations with hypertension, high cholesterol, diabetes, and stroke.

The walkability index was positively associated with five health outcomes. In addition, the coefficients of interaction terms between racial segregation variables and the walkability index show that different racial/ethnic population percentages have different effects on the associations between health outcomes and walkability: both the white population percentage and the black population percentage have reduced the positive association effects of walkability toward all five health outcomes; the minority population percentage has reduced the positive association effects of walkability toward hypertension and high cholesterol; the Asian population percentage has reduced the positive association effects of walkability toward high cholesterol.

The diagnostics results for spatial dependence of health outcomes in Table 1 indicate that SLM and SEM models are performed to improve prediction. The spatial regression model results in Table 4 indicate that the racial segregation index was positively associated with only one of five health outcomes, stroke. Compared to OLS models showing no associations between racial segregation and health outcomes, the spatial regression models show improved prediction by finding the positive association between the racial segregation index and stroke. For different racial/ethnic groups, the White population percentages have negative associations with hypertension, diabetes, coronary heart disease, and stroke; the Black population percentages have negative associations with diabetes and stroke; the Asian population percentages have a negative association with hypertension, but positive associations with diabetes and stroke; and the Minority population percentages have the positive association with hypertension, but negative associations with diabetes and stroke. In addition, the walkability variable is not significant for CVDs.

In addition, the coefficients of interaction terms between racial segregation variables and the walkability index show that racial segregation increased the negative association effects of walkability toward hypertension and stroke. Besides, the effects of different racial/ethnic population percentages on the associations between health outcomes and walkability varied: the White population percentage has reduced the negative association effects of walkability toward hypertension, diabetes, and stroke, but increased the negative association effects of walkability toward coronary heart disease; the Black population percentage has reduced the negative association effects of walkability toward diabetes and stroke; the Minority population percentage has reduced the negative association effects of walkability toward diabetes and stroke, but increased the negative association effects of walkability toward hypertension; the Asian population percentage has reduced the negative association effects of walkability toward stroke.

## 4. Discussion

The present research is one of a limited number of studies that explore the relationships of racial segregation with five health outcomes and walkability among adults older than 18 years old in the Chicago metropolitan area. The findings revealed the varied associations of different social segregation factors with each health outcome: among all racial segregations, the black population share has the largest effect on hypertension, diabetes, and stroke incidences, while the white population share has the largest effect on high cholesterol incidences and coronary heart disease incidences. Given the distinct histories of segregation in Chicago, these findings confirm that the distinct historical and social circumstances of segregation among blacks have shaped individual and community health in the literature [51]. The present findings may have significant implications for the inconsistency in the associations of racial segregation with health outcomes between racially segregated cities and other cities.

To the author’s knowledge, the previous literature does not provide much evidence of the moderating effects of racial segregation on the associations of health outcomes with walkability and the potential mechanisms behind these effects. The results of this study contribute to the literature on both urban design and health research by identifying racial segregation as the key moderator of the relationship between walkability and health outcomes. According to the OLS results, the impact of social segregation factors may be particularly important for the relationship between walkability and health outcomes. In general, social segregation factors reduce the positive associations between walkability and health outcomes, especially for the white population and the black population. Specifically, both the increased white and African American population shares reduced the positive associations between walkability and all health outcomes. These results could also be attributed to lifestyle behaviors resulting from the highly social segregation environments, such as physical activity, driving, and eating, which are strongly associated with health outcomes [52,53,54]. This suggests that the direct role of walkability in health outcomes might be affected by the social segregation environments in the context of Chicago. Future research in different social segregation contexts should be conducted to test these hypotheses.

The findings of CVDs following spatial regression analysis show that CVDs did not have any significant associations with walkability. This finding did not support the hypothesis that the areas with more walkable environments have a lower CVDs incidence than those areas with less walkable environments. This implies the existence of very important variables that affect CVDs other than the neighborhood walkability level. Besides, when considering the walkability level as a factor influencing CVDs, it is difficult to encapsulate the entire population in Chicago due to the high racial segregation in the city. The moderating effects of racial segregation on different CVDs’ health outcomes indicate the influence of neighborhood walkability on CVDs varied among racial/ethnic groups: the Black population faces disadvantages in benefiting from walkable environments compared to the White population, the Asian population, and other minority populations. Since the historical disinvestment in segregated neighborhoods could reduce the availability and accessibility of walkable neighborhood amenities [55], people must have a destination to walk to and a way to get to the destination to have walking activities, so accessibility is a prerequisite for walking behavior [56]. Fewer amenities’ destinations and/or less diverse land use in Black communities compared to other racial/ethnic communities may explain their disadvantages in walkable environments. Thus, further research to explore how exogenous factors closely tied to accessibility including connectivity, diversity of uses [57], topography, climate, and weather [58,59,60] can influence walking is warranted [61].

There are several limitations in the present study that must be considered. First, because of the cross-sectional design, this study was unable to determine the causality between variables. Second, the model-derived estimates from the 500 Cities Project can be affected by biases from both the survey itself (e.g., recall) and the modeling process, such as the choice of covariables [62,63]. Third, the walkability data from the SLD may not capture all relevant environmental features, such as sidewalk availability and park amenities that might promote activity [64]. Fourth, the aggregation level is based on data availability, so there is a boundary issue. Fifth, the unequal distribution of health outcomes might be caused by unobserved sociodemographic and economic characteristics, such as social vulnerability and air quality since these features might affect health outcomes [65,66,67]. Future studies examining the associations between social and environmental characteristics and health outcomes are warranted.

## 5. Conclusions

In conclusion, both social segregation factors and walkability were positively associated with all health-outcome incidences including hypertension, high cholesterol, diabetes, coronary heart disease, and stroke. In addition, social segregation reduced the positive associations of walkability with health outcomes, especially for the white and African American populations. These results are important in terms of informing public health policymakers and urban designers that more highly walkable neighborhoods may not necessarily facilitate health outcomes among African Americans and other minority populations.

## Figures and Tables

**Table 1:** Diagnostics for spatial dependence of health outcomes.

Diagnostics for Spatial Dependence	Hypertension	High Cholesterol	Diabetes	Coronary Heart Disease	Stroke
Lagrange Multiplier (lag)	<0.0001	<0.0001	<0.0001	>0.9999	<0.0001
Robust LM (lag)	<0.0001	>0.9999	<0.0001	>0.9999	<0.0001
Lagrange Multiplier (error)	<0.0001	<0.0001	<0.0001	<0.0001	<0.0001
Robust LM (error)	<0.0001	>0.9999	0.0014	<0.0001	<0.0001
Lagrange Multiplier (SARMA)	<0.0001	>0.9999	<0.0001	<0.0001	<0.0001

**Table 2:** Descriptive characteristics of health outcomes, racial segregation, and walkability.

Variables	Mean	Standard Deviation	Variance	Median	Min	Max
Hypertension	94.24447	86.33329	0.91606	71.16621	16.60493	1814.346
High cholesterol	261.0536	175.9568	0.67402	216.2422	65.43461	3628.692
Diabetes	35.49815	13.78195	0.38824	30.79028	3.42114	99.21314
Coronary Heart disease	17.49833	5.53586	0.31637	17.10571	3.42114	47.896
Stroke	10.86842	5.12221	0.47129	10.26343	3.42114	41.05371
Racial segregation	0.18234	0.09647	0.52909	0.18	0	0.7
White %	0.57041	0.32556	0.57075	0.67594	0	0.99225
Black %	0.19349	0.30689	1.58607	0.04131	0	1
Asian %	0.05953	0.08605	1.44545	0.02553	0	0.86334
Minority %	0.46216	0.34266	0.74143	0.37843	0	1
Walkability	11.54278	4.53846	0.39319	12.94445	0	19.66667

**Table 3:** Regression model results of health outcomes, racial segregation, and walkability in the Chicago metropolitan area.

	Hypertension	High Cholesterol	Diabetes	Coronary Heart Disease	Stroke
	Coef.	Coef.	Coef.	Coef.	Coef.
Racial segregation	2.27434	−1.31857	−0.86441	1.38322	−0.13146
White %	31.08971 ***	84.05319 ***	9.64448 ***	5.51958 ***	2.79960 ***
Black %	32.2922 ***	67.79394 ***	10.18328 ***	4.55355 ***	3.21920 ***
Asian %	16.24778	76.50946 ***	−1.85750	2.82629	0.62928
Minority %	5.85528 ***	20.3474 ***	2.40562 ***	0.44540	0.67025 ***
Racial segregation * Walkability	−0.13991	−0.05534	0.11981	−0.09103	0.02565
White % * Walkability	−2.36311 ***	−5.99343 ***	−0.78551 ***	−0.40177 ***	−0.22133 ***
Black % * Walkability	−1.30732 ***	−4.53305 ***	−0.34898 ***	−0.19644 ***	−0.04862 *
Asian % * Walkability	−1.09273	−5.34539 ***	0.16644	−0.15835	−0.03891
Minority % * Walkability	−0.330878 ***	−1.73176 ***	0.00214	−0.00492	−0.02207
Walkability	1.92689 ***	6.09360 ***	0.62089 ***	0.31459 ***	0.18598 ***
Less than high school percent	−1.03361	−4.39992 ***	1.67071 ***	0.19021	0.03403
Percentage of over 64	8.27113 ***	3.85662 **	2.61200 ***	2.83123 ***	1.35140 ***
No car percent	2.60904 ***	−1.96525 ***	2.25393 ***	1.13349 ***	1.10646 ***
Constant	0.77523 **	2.03459 ***	0.24136	0.12963	0.07547

* *p* ≤ 0.1; ** *p* ≤ 0.05; *** *p* ≤ 0.01.

**Table 4:** Spatial regression model results of health outcomes, racial segregation, and walkability in the Chicago metropolitan area.

	Hypertension	High Cholesterol	Diabetes	Coronary Heart Disease	Stroke
	Coef.	Coef.	Coef.	Coef.	Coef.
Racial segregation	26,727,056	548,942,642	114,122,549	1,416,647	92,526,221 ***
White %	−312,683,573 ***	320,663,413	−116,802,260 ***	2,417,411 **	−56,295,518 ***
Black %	6,806,294	−556,885,317	−278,219,034 ***	−188,056	−115,513,983 ***
Asian %	−457,509,550 **	968,993,480	145,575,641 ***	−1,813,597	144,240,748 ***
Minor %	392,657,293 ***	102,721,352	−171,964,215 ***	438,519	−179,778,592 ***
RS * Walkability	−1,967,636	−40,412,952	−8,401,659	−104,293	−6,811,746 ***
White % * alkability	23,019,649 ***	−23,607,121	8,598,939 **	−177,969 ***	4,144,455 ***
Black % * Walkability	−501,077	40,997,691	20,482,382 ***	13,845	8,504,097 ***
Asian % * Walkability	33,681,683 **	−71,336,940	−10,717,225 ***	133,516	−10,618,950 ***
Minority % * Walkability	−28,907,284 ***	−7,562,307	12,659,942 ***	−32,284	13,235,233 ***
Walkability	−0.1377830	−0.1901023	−0.1096064	−0.0174034	−0.0422031
CONSTANT	7	18	12	3	3

* *p* ≤ 0.1; ** *p* ≤ 0.05; *** *p* ≤ 0.01.

## Data Availability

Not applicable.
